# Exosomes derived from human adipose tissue-derived mesenchymal stem cells alleviate atopic dermatitis

**DOI:** 10.1186/s13287-018-0939-5

**Published:** 2018-07-11

**Authors:** Byong Seung Cho, Jin Ock Kim, Dae Hyun Ha, Yong Weon Yi

**Affiliations:** ExoCoBio Exosome Institute (EEI), ExoCoBio Inc., STE 306, 19 Gasan digital 1-ro, Geumcheon-gu, Seoul, 08594 Republic of Korea

**Keywords:** Exosome, Adipose tissue-derived mesenchymal stem cells, Atopic dermatitis, Inflammation

## Abstract

Exosomes are nano-sized vesicles (30–200 nm) constantly released by almost all cells. The ability of exosomes to travel between cells and deliver their cargo, which includes lipids, proteins, and nucleic acids, makes them an appealing cell-free therapy option to treat multiple diseases. Here, we investigated for the first time whether human adipose tissue-derived mesenchymal stem cell-derived exosomes (ASC-exosomes) can ameliorate atopic dermatitis (AD) in an in vivo mouse model. When injected either intravenously (IV) or subcutaneously (SC) into NC/Nga mice treated with house dust mite antigens, ASC-exosomes were found to reduce pathological symptoms such as clinical score, the levels of serum IgE, the number of eosinophils in blood, and the infiltration of mast cells, CD86+, and CD206+ cells in skin lesions. ASC-exosomes also significantly reduced mRNA expression of various inflammatory cytokines such as interleukin (IL)-4, IL-23, IL-31, and tumor necrosis factor-α (TNF-α) in AD skin lesions of Nc/Nga mice. Taken together, these results suggest that ASC-exosomes can be a novel promising cell-free therapeutic modality for AD treatment.

**Electronic supplementary material:**

The online version of this article (10.1186/s13287-018-0939-5) contains supplementary material, which is available to authorized users.

## Introduction

Since current treatment options for atopic dermatitis (AD) are limited and have potentially harmful side effects, there are unmet needs to develop novel therapies that are safe and efficacious [1]. Several biologics targeting pro-inflammatory cytokines are currently under development and dupilumab, a dual inhibitor of IL-4 and IL-13, was recently approved by the US FDA for treating adults with moderate to severe AD [1]. Although long-term follow-up study is needed to determine late side effects of dupilumab [1], its efficacy indicates that multiple targeting is a plausible way to treat AD [2].

Several studies have demonstrated that the allergic progress in AD could be suppressed by mesenchymal stem cells (MSCs) derived from human umbilical cord blood (UCB-MSC), bone marrow (BM-MSC), or adipose tissue (ASC) by modulating multiple targets [3]. However, therapeutic use of MSCs has several drawbacks, such as poor engraftment efficiency, potential tumor formation, unwanted immune responses, non-specific differentiation, short half-life, and the difficulty of quality control before administration [4].

Exosomes are nanovesicles (30–200 nm) released by almost all cells and found in all body fluids [4]. Exosomes deliver their cargo (proteins, lipids, and nucleic acids) from originating cells to recipient cells. Growing evidence suggests that exosomes derived from stem cells could be a promising alternative to cell-based therapy because exosomes would avoid most of the problems associated with cell-based therapy while recapitulating the therapeutic efficacy of stem cells [4]. For example, exosomes have no risk of tumor formation as they cannot replicate. They also can be sterilized by filtration and have a longer shelf-life than cells themselves. Being much smaller than stem cells, exosomes easily circulate through the body and reach sites of injury. In addition, long-term repetitive administration of exosomes does not elicit toxicity [5]. Here, we for the first time investigated the therapeutic effect of exosomes derived from human ASC (ASC-exosomes) on AD in a mouse model.

## Results and discussion

ASC-exosomes were isolated by a sequential filtration method from serum-free conditioned media (Additional file 1: Materials and methods) of ASCs and characterized according to the recommendation of the International Society for Extracellular Vesicles (ISEV) [6]. Transmission electron microscopy analysis and nanoparticle tracking analysis revealed the size distribution and number of ASC-exosomes (Additional file 1: Figure S1A, B). The characteristics of ASC-exosomes were also validated by western blotting with antibodies against surface markers (CD9, CD63, and CD81) and an internal marker (TSG101) (Additional file 1: Figure S1C). CD63 and CD81 were further analyzed by flow cytometry (Additional file 1: Figure S1D). We also used a cell-based assay as an in vitro potency assay for isolated ASC-exosomes. Stimulation of murine macrophage RAW264.7 cells with lipopolysaccharide (LPS) for 24 h substantially evoked production of nitric oxide (NO). However, co-treatment of ASC-exosomes significantly attenuated NO production in a dose-dependent manner (Additional file 1: Figure S2). Notably, the effect of ASC-exosomes was comparable to that of dexamethasone.

**Fig. 1:**
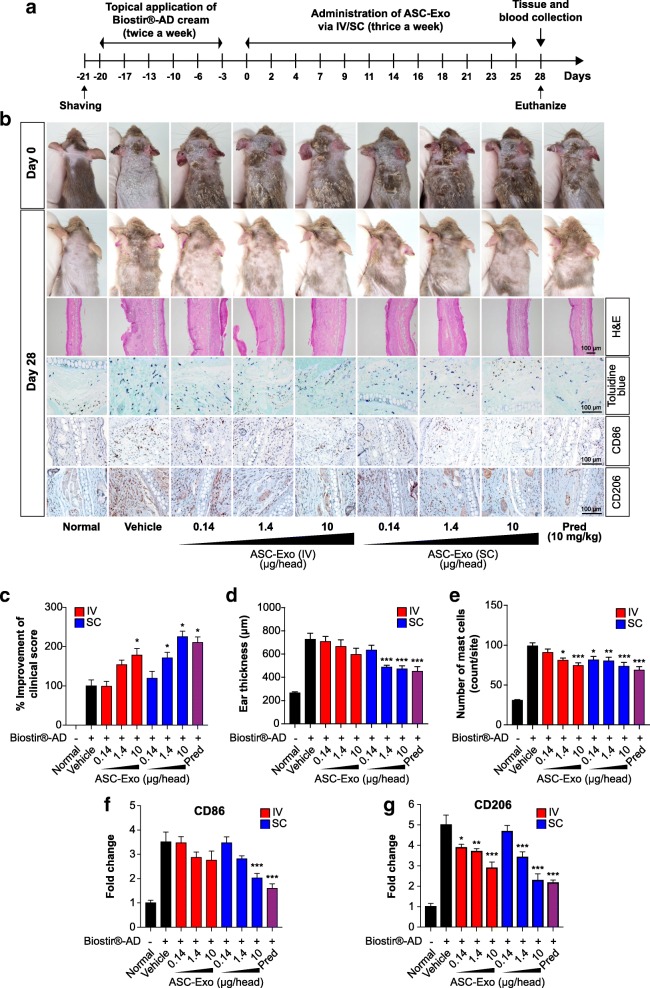
Therapeutic effects of ASC-exosomes on AD-like lesions in NC/Nga mice. **a** The study protocol. **b** Representative skin manifestations in NC/Nga mice at days 0 and 28, H&E staining results, toluidine blue staining results, and immunohistochemical staining of CD86+ or CD206+ cells of ear skin samples from AD mice. Enlarged images of toluidine blue staining for mast cells and immunostaining for CD86 and CD206 are shown in Additional file 1: Figures S4–S6, respectively. Indicated amounts of ASC-exosomes (micrograms/head) were administered either by IV or SC thrice a week for 4 weeks. **c** Relative percentage improvement of clinical skin severity scores compared to vehicle group. Percentage improvement was calculated as described in Additional file 1: Materials and methods. **d** Improvement of ear thickness as measured in H&E-stained tissue sections in **b**. **e** The number of mast cells in the skin lesions determined in toluidine blue-stained tissue sections in **b**. Quantitative analysis of CD86+ (**f**) and CD206+ (**g**) cells as determined in tissue sections in **b**. Results are presented as mean ± standard error of the mean; *n* = 10 for each group. *P < 0.05, **P < 0.01, and ***P < 0.001 vs vehicle control group. *IV* intravenous administration, *SC* subcutaneous administration, *Pred* prednisolone

To investigate whether ASC-exosomes ameliorate AD symptoms in vivo, we evaluated the effects of ASC-exosomes in a murine model. AD-like lesions were induced by Biostir®-AD cream, which contains antigens from house dust mite, in NC/Nga mice (Additional file 1: Materials and methods) and ASC-exosomes were administered either intravenously (IV) or subcutaneously (SC) thrice a week for 4 weeks (Fig. 1a). As a positive control, prednisolone (10 mg/kg) was orally administered daily. We found that both IV and SC administration of ASC-exosomes significantly decreased AD symptoms in a dose-dependent manner (Fig. 1b, c). Consistently, ear thickness was also reduced in ASC-exosome-treated mice (Fig. 1b, d). We also found that the number of infiltrated mast cells was significantly reduced by ASC-exosome administration (Fig. 1b, e and Additional file 1: Figure S4). Additionally, the numbers of CD86+ and CD206+ cells decreased in the skin lesions after ASC-exosome administration (Fig. 1b, f, g, Additional file 1: Figures S5 and S6). Interestingly, it has been reported that inflammatory dendritic epidermal cells (IDECs), which are not found in normal skin but are abundant in AD skin, express both CD86 and CD206 on their surfaces [7, 8].

**Fig. 2:**
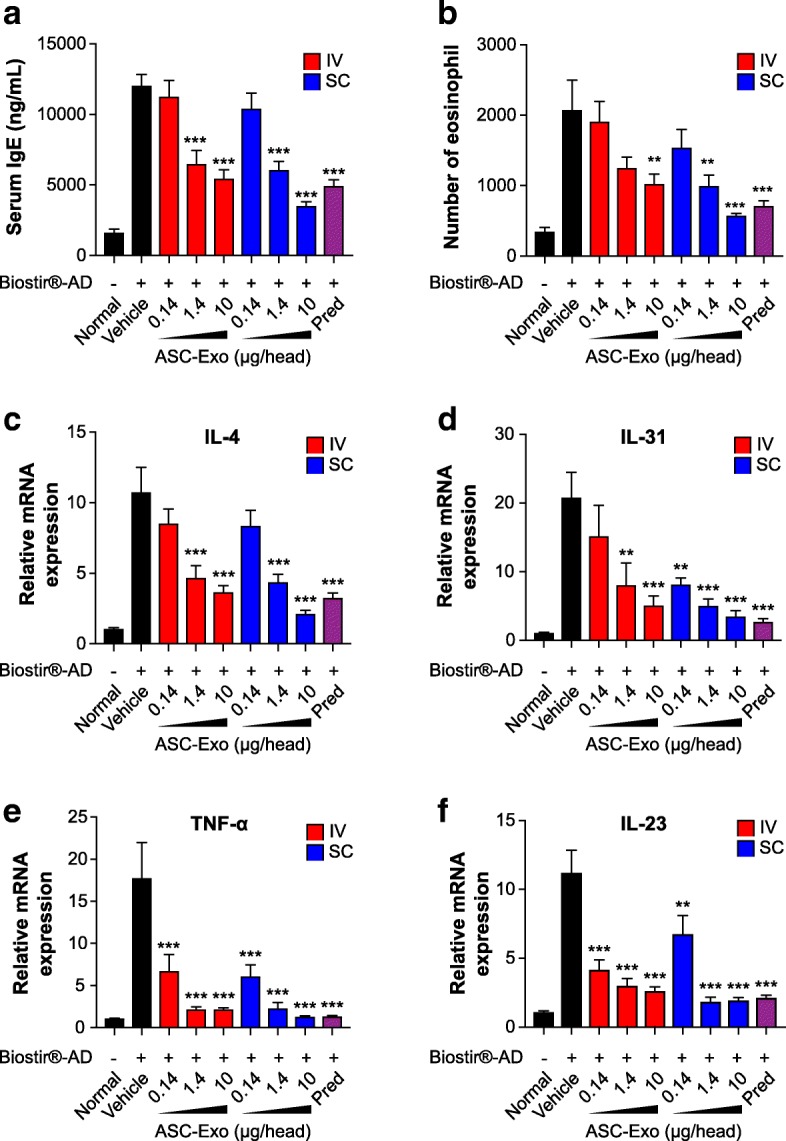
The effect of ASC-exosomes on the level of serum IgE, the number of eosinophils, and the expression of pro-inflammatory cytokines in NC/Nga mice. **a** The level of serum IgE was detected by ELISA. **b** The number of eosinophils in blood was determined by differential cell counting. **c**–**f** Total RNA was isolated and the mRNA levels of IL-4 (**c**), IL-31 (**d**), TNF-α (**e**), and IL-23 (**f**) were detected by quantitative real-time PCR and normalized by GAPDH mRNA expression. Results are presented as mean ± standard error of the mean; *n* = 10. *P < 0.05, **P < 0.01, and ***P < 0.001 vs vehicle group. *IV* intravenous administration, *SC* subcutaneous administration, *Pred* prednisolone

Since the elevation of the serum IgE level correlates with the severity of AD [9], we examined the effects of ASC-exosomes on the serum IgE level. The results showed that serum IgE levels were markedly reduced after ASC-exosome administration, either IV or SC, in a dose-dependent manner and these effects were comparable to that of prednisolone (Fig. 2a). As IgE mediates activation of mast cells and eosinophils [9], lowering serum IgE level by ASC-exosomes might result in reductions in mast cell infiltration (Fig. 1e) and the number of eosinophils. As expected, ASC-exosomes significantly lowered the number of eosinophils (Fig. 2b) but had little or no effect on neutrophil or white blood cell numbers (Additional file 1: Figure S3).

The mRNA levels of inflammatory cytokines were analyzed by quantitative real-time PCR (qRT-PCR). Interestingly, systemic administration of ASC-exosomes dose-dependently reduced the up-regulated mRNA levels of IL-4, IL-31, IL-23, and TNF-α in the skin lesions compared to vehicle control; the reduction was comparable to that with prednisolone treatment (Fig. 2c–f). In fact, all these pro-inflammatory cytokines are targets for biologics currently being developed or recently approved [1]. Downregulation of these multiple targets are well correlated with alleviation of AD symptoms in this study since IL-4 initiates isotype class switching to IgE and activates eosinophils [10]; IL-31 influences isotype class switching to IgE and recruits inflammatory cells into the skin and its level correlates with severity of AD [11]; IL-23 induces the differentiation of naïve T cells into highly pathogenic helper T cells that produce TNF-α [12]; and the plasma concentration of TNF-α is correlated with the severity of AD [13]. Collectively, these data demonstrate that systemic administration of ASC-exosomes ameliorates AD-like symptoms through the regulation of inflammatory responses and expression of inflammatory cytokines. These findings indicate that ASC-exosomes could be a novel cell-free therapeutic strategy to treat AD.

Despite the relevance of our findings, a limitation of the study is that potential donor variability remains to be addressed. In fact, a report has shown that donor age negatively impacts immuno-modulatory properties of ASC [14]. Further studies will be needed to confirm whether the potency of ASC-exosomes from aged donors correlates with our observations in younger, healthy donors.

## Additional file

Additional file 1: Supporting information. (DOCX 6577 kb)

## Abbreviations

AD: Atopic dermatitis
ASC: Adipose tissue-derived mesenchymal stem cell
BM: Bone marrow
IL: Interleukin
MSCs: Mesenchymal stem cells
qRT-PCR: Quantitative real-time PCR
TNF-α: Tumor necrosis factor-alpha
UCB: Umbilical cord blood
