# Supplementary material for: Exosomes derived from human adipose tissue-derived mesenchymal stem cells alleviate atopic dermatitis
Source: Stem Cell Res Ther. 2018 Jul 11;9:187. doi: 10.1186/s13287-018-0939-5 (PMC6042362; doi:10.1186/s13287-018-0939-5)
Supplement: Supplementary file 1 — Supporting information. (DOCX 6577 kb) [file 13287_2018_939_MOESM1_ESM.docx]

**Additional file 1**

**Exosomes derived from human adipose tissue-derived mesenchymal stem cells alleviate atopic dermatitis**

Byong Seung Cho^1^, Jin Ock Kim^1^, Dae Hyun Ha^1^, and Yong Weon Yi^1^*

^1^ExoCoBio Exosome Institute (EEI), ExoCoBio Inc, Seoul, 08594, Korea

*Corresponding author

E-mail: [cto@exocobio.com](mailto:cto@exocobio.com)

## Materials and Methods

**Isolation of exosomes from human ASCs**

Human ASCs were isolated from a 29-year-old healthy female donor and maintained in DMEM containing 10% fetal bovine serum (FBS) and 1% penicillin/streptomycin (Thermo Fisher Scientific; Carlsbad, CA). Exosomes were purified from the conditioned media of ASCs by a sequential filtration method. In brief, conditioned media were collected from ASCs (within passage 9) cultured in serum-free medium at 37 °C in a humidified atmosphere of 5% CO_2_ for 24 – 48 h. Cells, debris, and other larger particles were removed by centrifugation and filtration through 0.22-μm filter. ASC-Exosomes were further concentrated and purified by tangential flow filtration with molecular weight cut-off of 300 or 500 kDa. The protein concentration of purified ASC-exosomes was determined by BCA protein assay (Thermo Fisher Scientific; Carlsbad, CA).

## Transmission electron microscopy (TEM)

ASC-Exosomes were fixed by 0.5% glutaraldehyde (Sigma Aldrich, St. Louis, MO) in PBS for 12 h at 4 °C and dehydrated by incubating with absolute ethanol for 10 min at room temperature. Fixed exosomes were transferred on to the Formvar-Carbon coated EM grid (TED PELLA Inc., Redding, CA) and air-dried for 5 to 10 min. Phosphotungstic acid solution (1%) was used as a contrasting agent. The grid was washed in a large volume of absolute ethanol, air dried for 5 to 10 min. TEM images were obtained by the JEM 2100F TEM (JEOL, Tokyo, Japan).

## Nanoparticle tracking analysis (NTA)

The NanoSight NS300 (Malvern Instruments, Malvern, United Kingdom), equipped with a 642 nm laser, was used to analyze ASC-exosomes. The NanoSight polystyrene latex calibration beads, 100 nm and 200 nm, were applied to check the instrument performance. To analyze diluted ASC-exosomes, the camera level was maintained at 14 and 30 videos of typically 10 seconds duration were taken, with a frame rate of 30 frames per second. Alternatively, at least 2,000 particles were tracked and analyzed to prevent statistically insignificant peaks in the size distribution (Coumans et al. 2017). Data was analyzed by NTA 3.0 software.

## Western blot analysis

To characterize exosome, western blotting was carried out for the exosome surface markers such as CD9, CD63, CD81 and an internal marker TSG101. Briefly, protein samples were prepared from isolated exosomes by lysis with RIPA buffer (Cell Signaling Technology, Danvers, MA) supplemented with the protease inhibitor cocktail (Roche, Mannheim, Germany). Total protein (40 μg) was subsequently resolved on a 12% polyacrylamide gel and transferred onto polyvinylidene fluoride (PVDF) membranes using semidry system. The membranes were blocked with 3% skim milk (BD Biosciences, Franklin, NJ) in TBST (0.1% Tween 20 in Tris-buffered saline; 137 mmol/L NaCl and 20 mmol/L Tris/HCl, pH 7.4) for 1 h at room temperature. Membranes were then incubated with primary antibodies at 4 °C overnight. Next, membranes were washed three times with TBST and further incubated with the appropriate horseradish peroxidase (HRP)-conjugated secondary antibodies (Jackson ImmunoResearch, West Grove, PA) for 1 h at room temperature. The blots were visualized by enhanced chemiluminescence (ECL) detection reagents (DoGenBio, Seoul, Korea) and exposed onto X-ray film. The following primary antibodies were used in this study: anti-CD9, anti-TSG-101 (Abcam, Cambridge, MA), anti-CD63, and anti-CD81 (Systems Biosciences, Palo Alto, CA).

## Flow cytometry

To capture CD63-positive exosomes, Dynabeads coated with human CD63 antibody (Thermo Fisher Scientific, Carlsbad, CA) was used according to manufacturer’s recommendation. In brief, ASC-exosomes in Isolation Buffer (0.1% BSA in PBS) were incubated with Dynabeads for overnight at 4 °C with gentle mixing. The next day, the bead-bound exosomes were washed by Isolation Buffer and placed on the magnet for 1 min to discard the supernatant. The bead-bound exosomes were resuspended in Isolation Buffer and incubated with PE-mouse anti-human CD63 or PE-mouse anti-human CD81 (BD Biosciences, San Jose, CA) for 1 hours at room temperature with gentle mixing. The labelled exosomes were washed with Isolation Buffer and analyzed using NovoCyte 2000® flow cytometer (ACEA Biosciences, San Diego, CA).

## Nitric oxide (NO) detection

The murine macrophage cell line RAW 264.7 were maintained in DMEM with 10% heat inactivated fetal bovine serum, 100 U/mL penicillin and 100 mg/mL streptomycin, under 5% CO_2_ at 37 °C, in humidified air. Cells were plated in 24-well plates. After 24 h, cells were stimulated by serum-free medium containing LPS (10 ng/mL) and co-treated with dexamethasone (50 μM) or exosomes. Supernatants were collected after 24 h treatment and centrifuged at 1500 rpm for 5 min to remove cells and debris. The amount of nitric oxide was determined by Nitric Oxide Detection Kit (iNtRON, Seongnam, Korea), based on diazotization (Griess method), according to manufacturer’s instruction. The signals were measured by a SpectraMax i3x microplate reader (Molecular Devices, Sunnyvale, CA).

## Induction of atopic dermatitis in mice

The animal study was approved by the Institutional Animal Care and Use Committee and performed in accordance with the Animal Experimentation Policy. AD-like skin lesions were induced in 5-week-old male NC/Nga mice (Central Lab. Animal Inc, Seoul, Korea) by applying Biostir®-AD Cream (Biostir, Inc., Osaka, Japan), an ointment containing allergens of the house dust mite (*D. farinae*), as described previously (Kim et al. 2013). Briefly, after removal of hair, 150 μL of 4% (w/v) sodium dodecyl sulfate was applied to the pinna area and dried by cold air. Biostir®-AD Cream was applied topically twice a week for 3 weeks. At the start of the experiment, mice were randomly divided into the following groups: normal (negative control), vehicle-treated, ASC-exosomes-treated via IV, ASC-exosomes-treated via SC, and prednisolone-treated (positive control). Three different doses of exosomes were administered: 0.14 μg/head, 1.4 μg/head, and 10 μg/head. Vehicle and ASC-exosomes were administered (by IV or SC) thrice a week for four weeks and prednisolone (10 mg/kg) was daily administered orally.

## Clinical examinations

The animals were housed in an air-conditioned room and maintained at temperature 23 ± 2°C, relative humidity 55 ± 10%, and lighting 12 h on/12 h off. Mice were weighed at the day of exosome administration and once a week and on the day of euthanasia. Clinical severity of lesions of mice were estimated using the scores of Matsuda (Suto et al. 1999). The total score for AD-like skin lesions was designated as the sum of individual scores graded as 0 (none), 1 (mild), 2 (moderate), and 3 (severe) for the symptoms of erythema/hemorrhage, edema, excoriation/erosion and scaling/dryness. To exclude the effects of vehicle, the relative percent improvement of clinical score (CS) compared with vehicle was calculated by [│CS_28_-CS_0_│/CS_0_]/[│CS_28v_-CS_0v_│/CS_0v_] × 100, where CS_0_ is CS of each mouse at day 0; CS_28_ is CS of each mouse at day 28; CS_0v_ is mean CS for vehicle control group at day 0; CS_28v_ is mean CS for vehicle control group at day 28.

## Histological Analysis

The tissues of the experimental mice were removed, fixed in 10% phosphate-buffered formalin, embedded in paraffin, sectioned, and stained with hematoxylin and eosin (H&E) solution. Thickness of tissue section was measured by Zen 2.3 Blue Edition (Car Zeiss, Goettingen, Germany) at three different sites in a randomly captured images of H&E staining. For the measurement of mast cell infiltration, the paraffin-embedded tissue sections were stained with toluidine blue and the number of mast cells was counted in five random sites. To detect CD86+ or CD206+ cells in the skin lesions, paraffin-embedded tissue sections were mounted on a glass slide, deparaffinized in xylene and rehydrated in a graded ethanol series. After washing with water, the sections were antigen-retrieved using citrate buffer (pH 6.0; DAKO) in a steamer for 20 minutes and cooled to room temperature. Sections were then washed with TBST, treated with 0.03% H_2_O_2_, blocked with 4% BSA and dextran, incubated with a primary antibody anti-CD86 (Abcam, Cambridge, MA) or anti-206 (Abcam, Cambridge, MA). Immunohistochemical staining was developed using the DAB substrate system (DAKO).

## Measurement of Serum IgE

Serum IgE level was determined by IgE ELISA Kit (Thermo Fisher Scientific) according to manufacturer’s instruction.

## Differential Cell Counting

Peripheral blood mononucleated cells (PBMCs) were isolated by Histopaque-1077 (Sigma Aldrich) according to manufacturer’s protocol. Isolated PBMCs were washed twice with PBS and resuspended in RPMI 1640 media (Thermo Fisher). The number of PBMCs were determined by TC-20 automated cell counter (Bio-Rad). One hundred μL of cells (total 1 x 10^5^ cells) were loaded onto Cytospin slide and centrifuged by Shadon Cytospin 4 (Thermo Fisher). PBMCs were dried, fixed by ethanol, and stained with Diff-Quik (Sigma Aldrich), and differentially counted in 5 different fields under 400x magnification.

## Real-time PCR

Total RNA was extracted from tissues by hybrid-R RNA Prep Kit (GeneAll Biotechnology Co., Ltd, Seoul, Korea) according to the manufacturer’s protocol. The concentration of RNA was measured by the Take3 Multi-Volume Plate (Biotek Instruments, Winooski, VT). The synthesis of cDNA was performed with ReverTra Ace qPCR RT Master Mix with gDNA Remover (Toyobo, Osaka, Japan). After cDNA synthesis, real-time PCR analysis was performed by CFX96 Touch Real-Time PCR Detection System (Bio-Rad, Hercules, CA) with WizPure qPCR Master Mix (Wizbiosolutions, Seongnam, Korea) and normalized to the mean of GAPDH. The primers used in this study are: IL-4, (forward) 5’-ACA GGA GAA GGG ACG CCA T-3’ and (reverse) 5’-GAA GCC CTA CAG ACG AGC TCA-3’; IL-31, (forward) 5’-CAC ACA GGA ACA ACG AAG CC-3’ and (reverse) 5’-CGA TAT TGG GGC ACC GAA G-3’; TNF-α, (forward) 5’- CGT CGT AGC AAA CCA CCA AG-3’ and (reverse) 5’- TTG AAG AGA ACC TGG GAG TAG ACA-3’; IL-23p19, (forward) 5’- CAC ATG CAC CAG CGG GAC AT-3’ and (reverse) 5’- CTT TGC AAG CAG AAC TGG CTG TTG-3’; GAPDH, (forward) 5’- CAT GGC CTT CCG TGT TCC TA-3’ and (reverse) 5’- CCT GCT TCA CCA CCT TCT TGA T-3’. All the primers were synthesized from Bioneer Corp (Daejeon, Korea).

## Statistical analysis

One-way ANOVA (for parametric multiple comparisons, with Dunnett’s multiple comparison test for *post hoc* comparison), Kruskal-Wallis’ H-test (for non-parametric comparisons, with Dunn’s multiple comparison test for *post hoc* comparison), and Student’s *t*-test were used for statistical analysis and performed using Prism 5.03 (GraphPad Software Inc., San Diego, CA).

## References

Coumans FAW, Brisson AR, Buzas EI, Dignat-George F, Drees EEE, El-Andaloussi S, et al. Methodological guidelines to study extracellular vesicles. Circ. Res. 2017. p. 1632–48

Kim MC, Lee CH, Yook TH. Effects of Anti-inflammatory and Rehmanniae radix Pharmacopuncture on Atopic Dermatitis in NC/Nga Mice. JAMS J. Acupunct. Meridian Stud. 2013;6(2):98–109

Suto H, Matsuda H, Mitsuishi K, Hira K, Uchida T, Unno T, et al. NC/Nga mice: A mouse model for atopic dermatitis. Int. Arch. Allergy Immunol. 1999. p. 70–5

**Supplemental Figures**

**
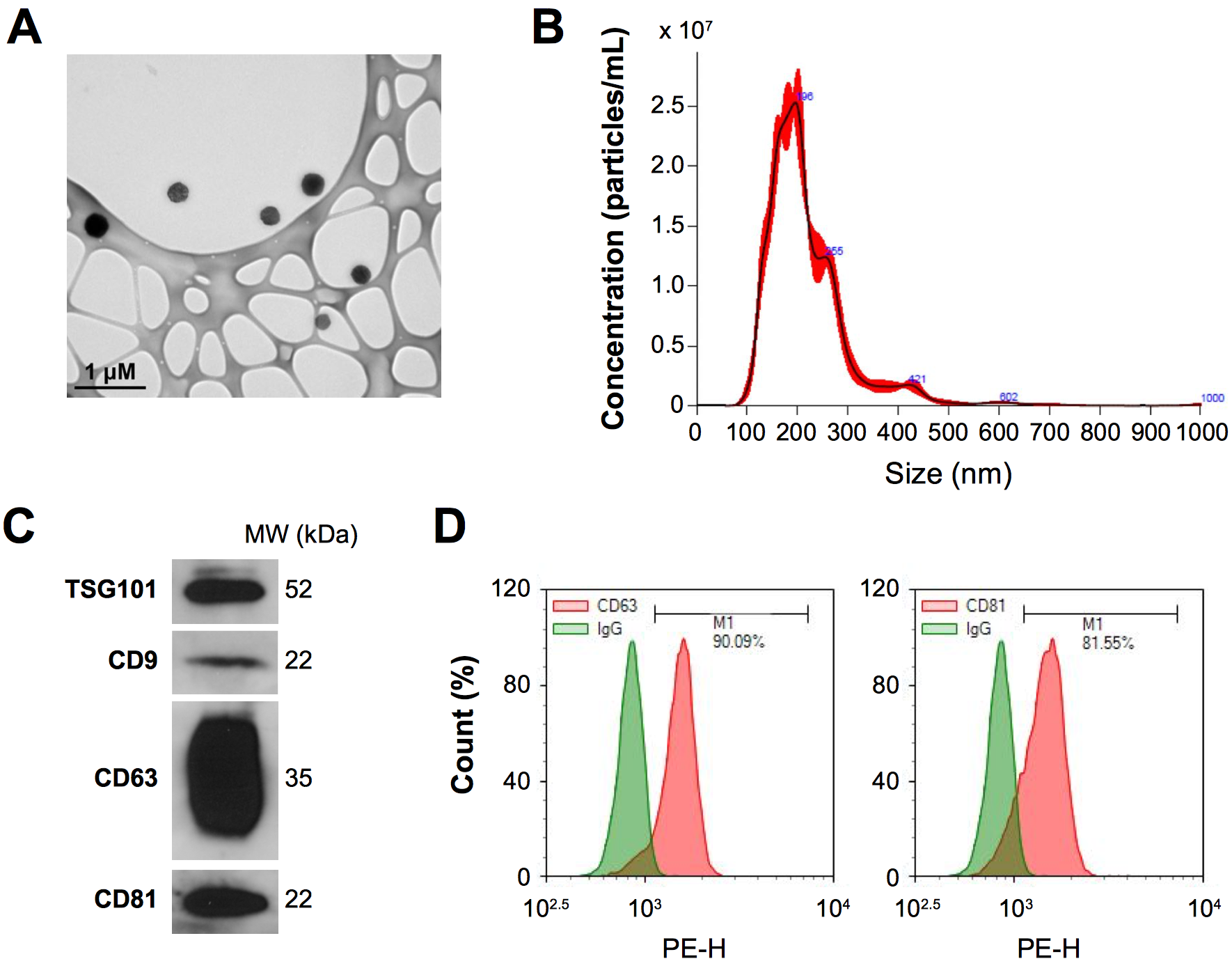
**

**Fig. S1. Characterization of isolated ASC-exosomes.** (A) Morphological characterization of ASC-exosomes by TEM analysis. Scale bar = 1 μm. (B) NTA histogram of ADC-exosomes. (C) Western blot analyses of exosome markers such as CD9, CD63, CD81 and TSG101. (D) Flow cytometric analysis of ASC-exosomes with antibodies against CD63 and CD81, respectively.


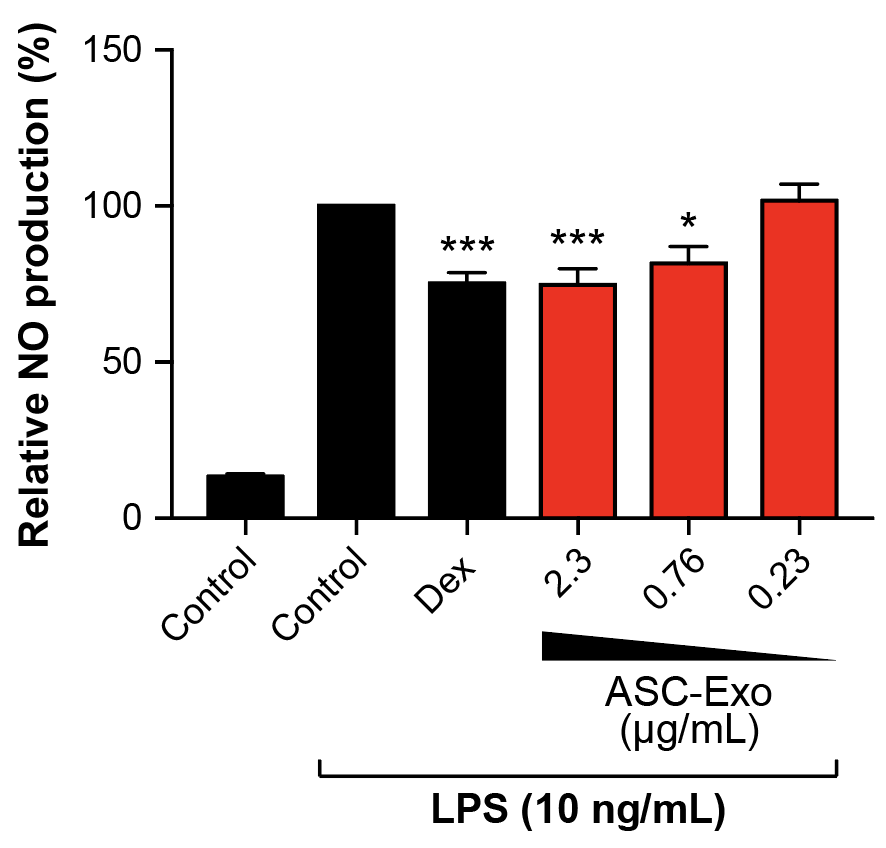


**Fig. S2. ASC-exosomes suppressed the LPS-induced responses in RAW264.7 macrophage.** (A) Effects of ASC-exosomes on the LPS-induced NO production in RAW264.7 cells. 100 μM of dexamethasone (Dex) was used as a positive control. Data are presented as mean ± SEM. n=6. **P* < 0.05 and ****P* < 0.001 vs control in the presence of LPS.

**
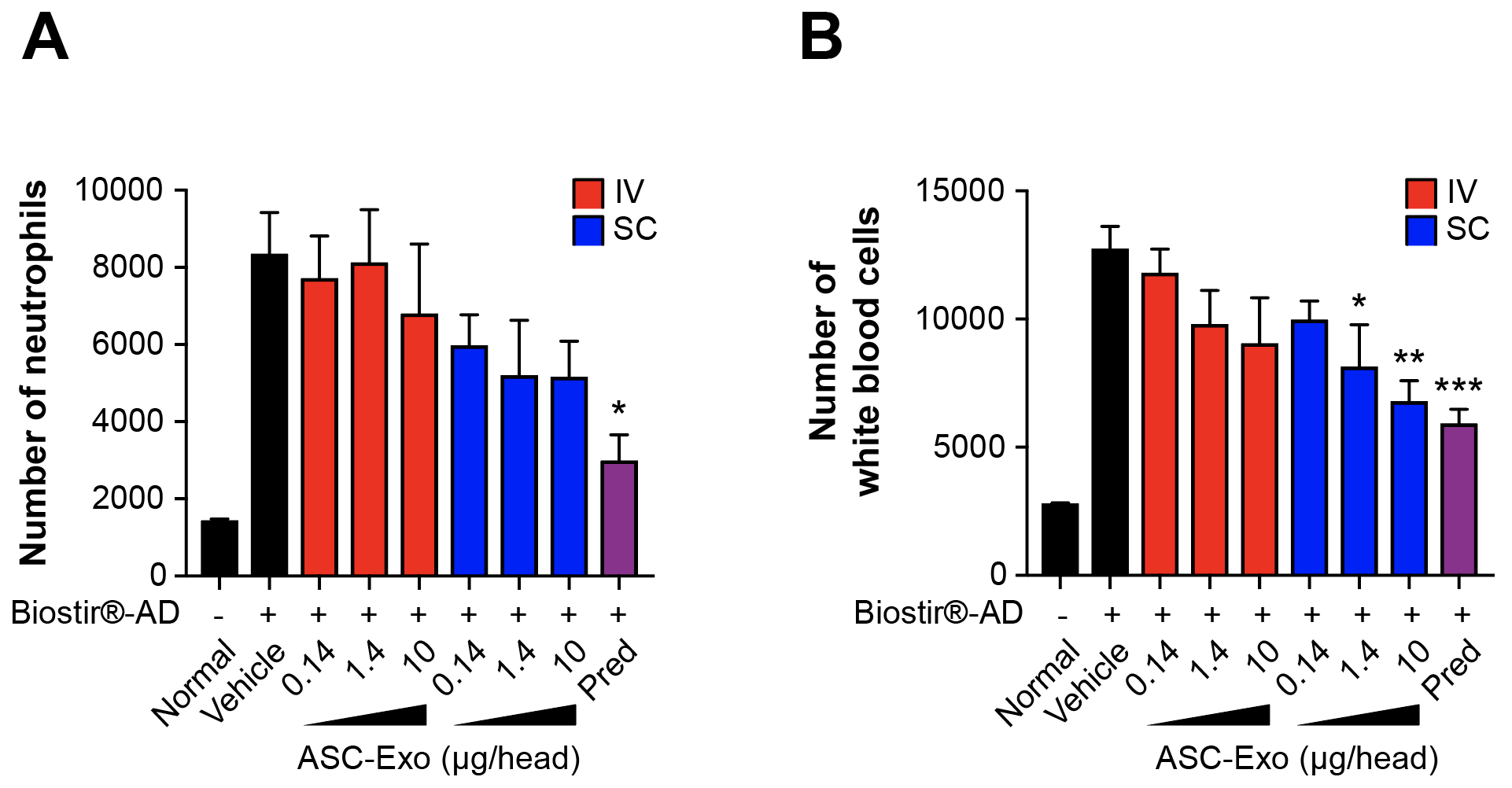
**

**Fig. S3. Effect of ASC-exosomes on the number of blood cells.** The number of neutrophils (A) and white blood cells (B) from the blood of AD mice. Data are presented as mean ± SEM. n = 10 for each group. **P* < 0.05, ***P* < 0.01 and ****P* < 0.001 vs vehicle control group. IV, intravenous; SC, subcutaneous; Pred, Prednisolone.

**
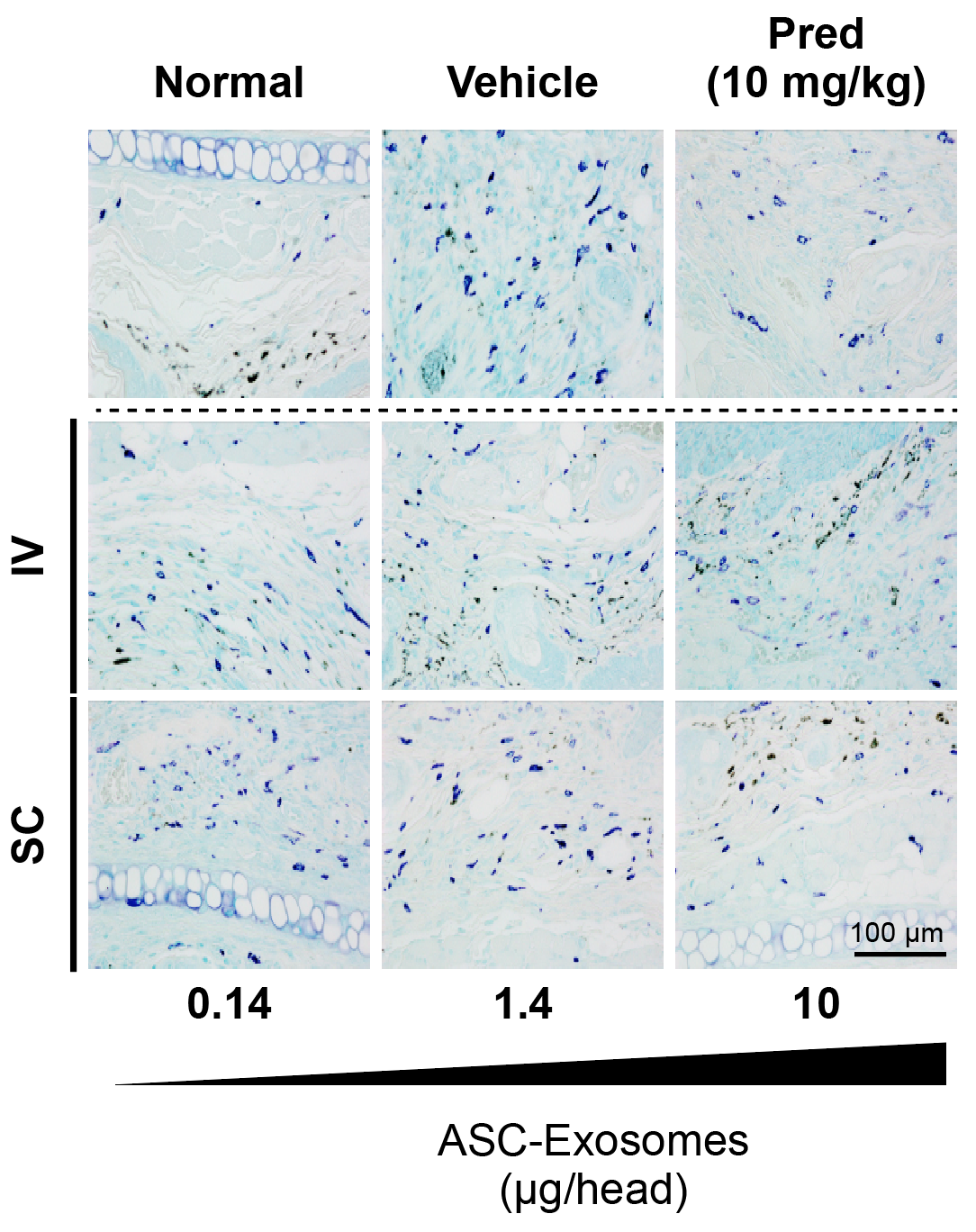
**

**Fig. S4. The enlarged images of toluidine blue staining for mast cells in the AD skin lesions shown in Fig. 1B.**

**
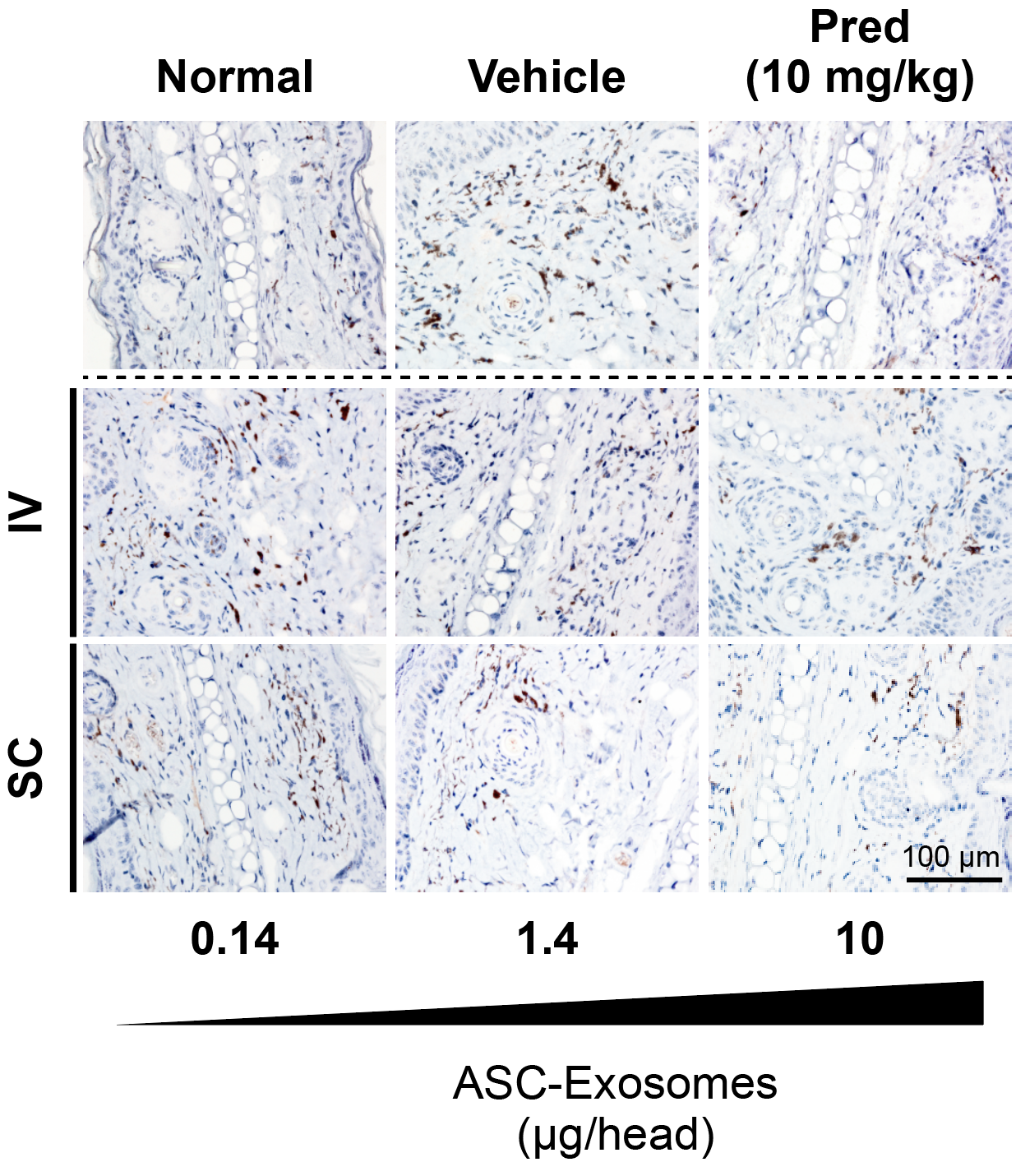
**

**Fig. S5. The enlarged images of immunohistochemical staining of CD86 in the AD skin lesions shown in Fig. 1B.**

**
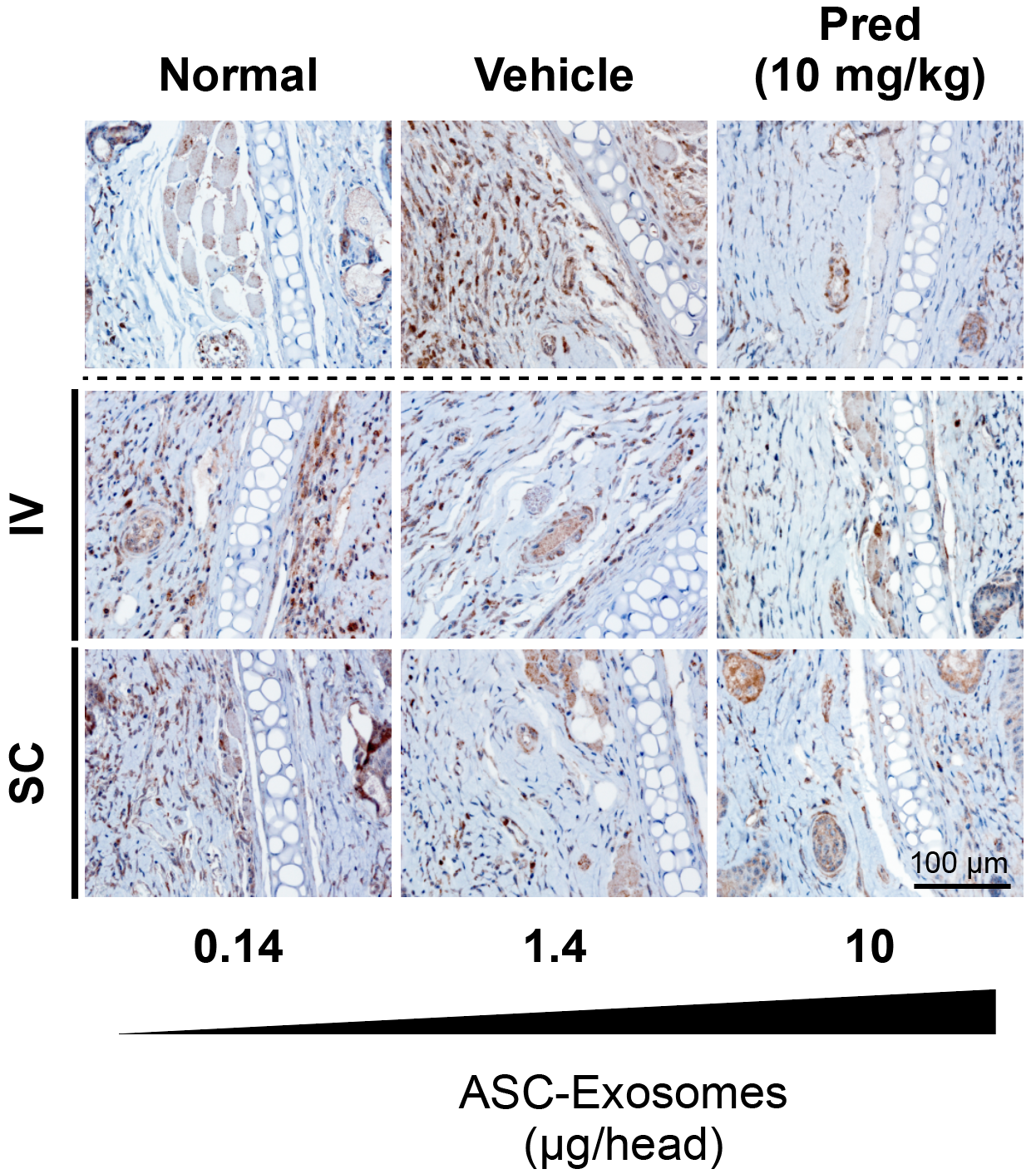
**

**Fig. S6. The enlarged images of immunohistochemical staining of CD206 in the AD skin lesions shown in Fig. 1B.**
